# Determination of Split Renal Function Using Dynamic CT-Angiography: Preliminary Results

**DOI:** 10.1371/journal.pone.0091774

**Published:** 2014-03-11

**Authors:** Andreas Helck, Ulf Schönermarck, Antje Habicht, Mike Notohamiprodjo, Manfred Stangl, Ernst Klotz, Konstantin Nikolaou, Christian la Fougère, Dirk Andrè Clevert, Maximilian Reiser, Christoph Becker

**Affiliations:** 1 Institute for Clinical Radiology, University Hospital Grosshadern (LMU), Munich, Germany; 2 Department of Internal Medicine IV, University Hospital Grosshadern (LMU), Munich, Germany; 3 Transplant Center Munich, University Hospital Grosshadern (LMU), Munich, Germany; 4 Department of Surgery, University Hospital Grosshadern (LMU), Munich, Germany; 5 Siemens Healthcare, Computed Tomography, Forchheim, Germany; 6 Department of Nuclear Medicine, University Hospital Grosshadern (LMU), Munich, Germany; University of Louisville, United States of America

## Abstract

**Objectives:**

To determine the feasibility of a dynamic CT angiography-protocol with regard to simultaneous assessment of renal anatomy and function.

**Methods:**

7 healthy potential kidney donors (58±7 years) underwent a dynamic computed tomography angiography (CTA) using a 128-slice CT-scanner with continuous bi-directional table movement, allowing the coverage of a scan range of 18 cm within 1.75 sec. Twelve scans of the kidneys (n = 14) were acquired every 3.5 seconds with the aim to simultaneously obtain CTA and renal function data. Image quality was assessed quantitatively (HU-measurements) and qualitatively (grade 1–4, 1 = best). The glomerular filtration rate (GFR) was calculated by a modified Patlak method and compared with the split renal function obtained with renal scintigraphy.

**Results:**

Mean maximum attenuation was 464±58 HU, 435±48 HU and 277±29 HU in the aorta, renal arteries, and renal veins, respectively. The abdominal aorta and all renal vessels were depicted excellently (grade 1.0). The image quality score for cortex differentiation was 1.6±0.49, for the renal parenchyma 2.4±0.49. GFR obtained from dynamic CTA correlated well with renal scintigraphy with a correlation coefficient of r = 0.84; P = 0.0002 (n = 14). The average absolute deviation was 1.6 mL/min. The average effective dose was 8.96 mSv.

**Conclusion:**

Comprehensive assessment of renal anatomy and function is feasible using a single dynamic CT angiography examination. The proposed protocol may help to improve management in case of asymmetric kidney function as well as to simplify evaluation of potential living kidney donors.

## Introduction

Assessment of accurate renal function as well as renal anatomy is necessary in the preoperative evaluation of potential living kidney donors, but also for other kidney diseases where knowledge of split renal function may guide optimal patient management. Estimation of glomerular filtration rate (GFR) from serum creatinine is fast, inexpensive, and widely accepted as a clinically useful parameter. However, creatinine-based formulas always apply to both kidneys together and therefore do not provide split renal function.

Determination of single-kidney-GFR of the affected as well as the contralateral kidney is essential for selecting the appropriate therapeutic regimen in case of unilateral kidney diseases like atrophy, obstruction or renal neoplasm [1]. While patients with vascular complications and mild impairment of renal function most likely benefit from revascularization [2] a mild decrease of the GFR of the affected kidney will not be detected by creatinine-based formulas. Moreover, in healthy subjects evaluated for potential kidney donation, determination of split renal function is required. Minimum single-kidney-GFR values in this context are recommended in order to avoid postoperative renal insufficiency for both the renal donor and the transplant recipient [3].

In addition to preinterventional assessment of the kidney anatomy using cross-sectional imaging, renal scintigraphy has to be applied separately to obtain split renal function, which is overall a relatively expensive and time-consuming practice.

Since dynamic CT angiography is feasible for simultaneous acquisition of morphological and functional information in renal transplants [4], we aimed to assess the feasibility of this promising technique to measure split renal function in subjects with native kidneys.

## Methods

### Patients

The study was designed as a prospective, non-randomized, clinically controlled cohort study. The study was approved by the local ethics committee (ethics committee of Ludwig Maximilians University, Project-Nr. 440-08) and all subjects provided written informed consent. During the study period 48 persons have been evaluated as potential renal donors at our hospital. Routinely total GFR was assessed by 24 h urine collection (which approximately reflects total GFR), for split renal function subsequently MAG3-scintigraphy was performed. To avoid unnecessary additional dose exposure DTPA-scintigraphy (which was an essential inclusion criterion for our study, since it allows for accurate assessment of GFR) has only been performed when 24 h urine collection was inconclusive (which was the case in 7 of 48 subjects).

### Scan protocol

The dynamic CTA was performed under free-breathing with a 128-slice CT scanner (Somatom Definition AS+; Siemens Healthcare, Germany) using 12 consecutive dynamic scans (80 kV/120 mAs) such that pre-contrast, arterial and venous phases were covered. The acquisition was executed with the adaptive 4D spiral mode of the scanner that allows continuous repetitive helical scanning of a range without stopping the table. The table is smoothly accelerated and decelerated without sudden starts or stops in order to minimize patient motion. The scan range was 18 cm, which were scanned in 1.75 s. The radiation was only turned on in the cranio-caudal motion phase resulting in a temporal sampling of 3.5 sec and a total examination time of about 40 s. 30 mL iodinated contrast medium (Imeron 400, Bracco Imaging GmbH, Konstanz/Germany) was injected via a cubital vein (flow rate 5 mL/s) using a CT power injector (Medrad Medizinische Systeme GmbH, Volkach/Germany) and a standard delay of 4 s. Subsequently 100 mL saline chaser (B. Braun Melsungen AG, Berlin/Germany) was injected with a flow rate of 3 mL/s. The examinations were performed by using a protocol with a collimation of 128×0.6 mm, the raw data were reconstructed to 3 mm sections (increment 1.5 mm) for the functional study and to 1.5 mm sections (increment 0.75 mm) for the morphological information respectively. The CT-examination was conducted 2 hours after the renal scintigraphy.

### Assessment of image quality

Post-processing and analysis was done on a dedicated workstation using a 3D software (MMWP, Siemens Healthcare, Germany) allowing to go through the dynamic phases in a cine mode or independently, phase by phase. For attenuation measurements (Hounsfield Units, HU) the optimal enhancement phases of the abdominal aorta, the renal artery, the renal parenchyma and the renal vein were selected. Size adapted ROIs were placed manually in the centre of the respective vessel. The maximum attenuation values (HU; mean and standard deviation) of the vessels were registered. Image noise was defined as the standard deviation of attenuation value measured in the foam mattress of the CT table (ROI was 100 mm^2^ in size). For calculation of the contrast-to-noise-ratio (CNR), HU values of the psoas muscle were measured in an identical manner. To minimize bias, 5 measurements were obtained in the mattress and psoas muscle per patient, and the mean of these values was used for further calculation. The CNR was calculated as CNR = (HU _aorta_ – HU _psoas muscle_)/Noise.

The quality of depiction of the renal artery, renal vein, cortex-medulla differentiation and renal parenchyma was rated in a consensus reading of 2 radiologists and appraised on the basis of a 4 point scale (1 = excellent, 2 = good, 3 = adequate, 4 = non diagnostic). Effective dose was calculated by multiplying the dose length product with a conversion factor of 0.015 [5].

### GFR calculation

**Figure 1:**
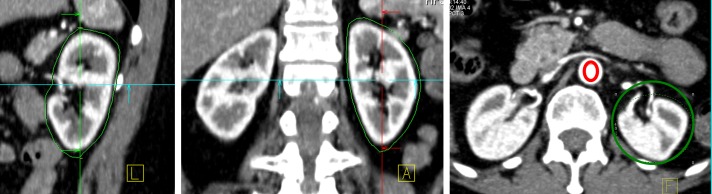
Semi-automatic segmentation of the left kidney and selection of a ROI in the aorta for the time attenuation curves (TACs) of the renal parenchyma and the arterial input.

Post-processing and analysis was performed on a dedicated workstation using the VPCT-Body software (Siemens Healthcare, Erlangen/Germany). Motion correction of the VPCT-Body software was used to minimize motion artifacts caused by respiration. In order to reduce the calculation time, the kidney was first outlined roughly in 3 orthogonal planes (Fig.1). The arterial input function (AIF) was taken from an oval region-of-interest (ROI) placed manually in the centre of the abdominal aorta (1.5 cm proximal to the outlet of the renal arteries) keeping an adequate distance from the vessel wall or calcified plaques (Fig.1 right).

**Figure 2:**
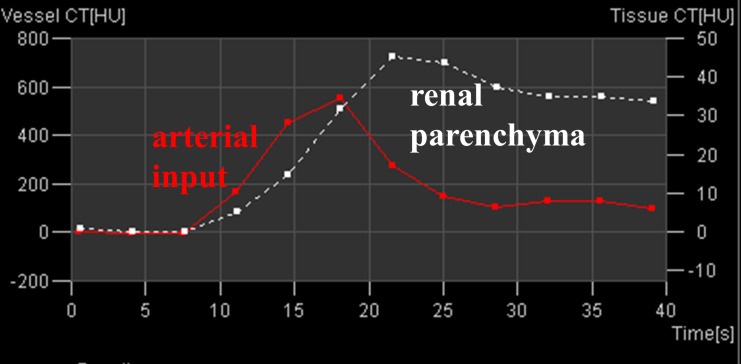
Time attenuation curves of the arterial input (red line) and of the renal parenchyma (white dashed line); note the different scale of the y-axis on the left (arterial input) and the right (renal parenchyma).

The AIF and the time-attenuation-curve (TAC) of the whole kidney were plotted (Fig 2) and checked for consistency before starting the voxel-based calculation.

The concentrations C(t) and C_A_(t) in tissue and artery, respectively, were calculated by subtracting the baseline signal from the time attentuation curves. Concentrations were fitted voxel-by-voxel according to the Patlak method [6]:
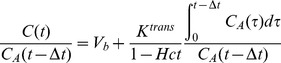
(1)


If the model is valid, a plot of the data for each t produces a straight line. V_b_ is the fractional blood volume (mL blood pro mL tissue), K^trans^ in the cortex is the glomerular filtration rate per unit of tissue volume (mL per min and per 100 mL tissue) [7] and Hct is the patients' haematocrit, which was determined from blood samples taken on the same day as the CT examination. The Patlak model in the renal cortex is valid only if the data used for the fit are restricted to the times before the tubular peak (so that tracer washout out of the tubuli is negligible) and after the vascular peak (so that bolus dispersion in the capillary bed is negligible) [8].

**Figure 3:**
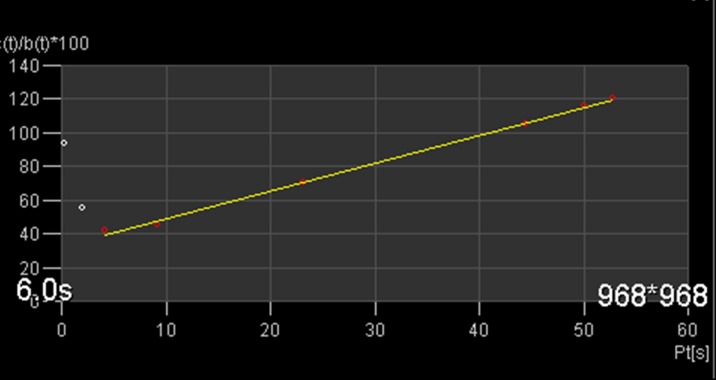
Patlak plot of a small ROI in the cortex: shown is the enhancement in the ROI relative to the enhancement in the aorta (c(t))/b(t)) as a function of the Patlak time (Pt) (see text for details). The selected points after the aortic peak (red points) fit very well by a straight line.

In order to maximally satisfy these assumptions, a Patlak start time was set manually to the time point immediately after the first-pass peak in the AIF, and only time points later than the chosen value were used in the fit. An end time was not set, as it can be safely assumed that no tracer leaves the proximal tubuli within a measurement window of 40 s. Typically 6 to 8 time points were used for the fit. The validity of the Patlak model in the chosen range was verified by inspecting the Patlak-plot for small ROIs in the cortex in each case (Fig.3) and verifying the linearity of the data.

The delay Δt between artery and tissue was determined separately for every voxel such that the cross correlation coefficient between the shifted AIF and the local TAC was maximal.

**Figure 4:**
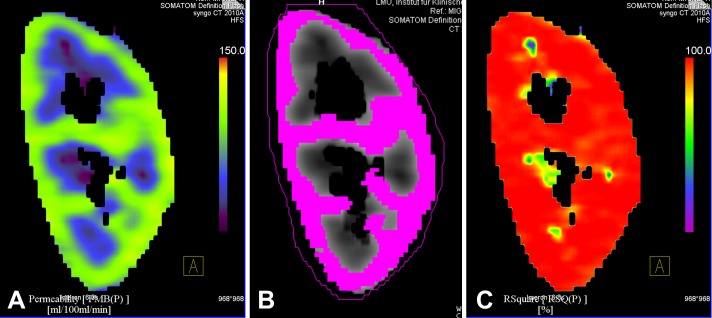
Demarcation of the operating renal cortex in a coronal reformation of the Ktrans maps (A). Cortex volume, calculated with a volume-program by setting upper and lower limits (upper limit 300 mL/100 mL/min, lower limit 80 mL/100 mL/min, volume 59 mL, mean Ktrans 98±10 (B). r2 map shows linearity of the Patlak fit in % (C).

On the generated K^trans^ maps the demarcation of the operating cortex became well recognizable (Fig.4 A), whereas the absolute value of K^trans^ varied depending on renal function. The K^trans^ maps were used to generate VOI based K^trans^ values of the operating kidney by loading the K^trans^ images in a volume-program (Volume, Siemens Healthcare, Erlangen/Germany). The lower threshold (the upper threshold was fixed at K^trans^ 300 mL/min/100 mL) was adjusted until the highlighted area agreed with the operating cortex demarcated in a central coronally reformated K^trans^ map (Fig.4 B). The volume program then allowed for determination of V_cortex_, average K^trans^ and its standard deviation for the whole kidney (Fig. 4). We checked whether there was a good fit of the Patlak plot by inspecting the r^2^ map (square of the correlation coefficient of the Patlak regression line for each voxel) in the same coronal reformation (Fig 4 C).

Finally, GFR was calculated from the average K^trans^ and the cortical volume derived from the VOI:
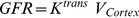
(2)


### Renal scintigraphy as reference standard for split renal function (SRF)

We compared our results with our standard of reference which was the combined assessment of GFR by means of plasma disappearance of [^99m^Tc]-labeled diethylenetriaminopentacetic acid (DTPA) and the evaluation of the relative renal function (RRF) by means of dynamic renal scintigraphy with [^99m^Tc]-labeled mercaptoacetyltriglycerine (MAG-3).
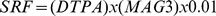



#### a) GFR-measurement using [^99m^Tc]-DTPA

Assessment of global GFR was routinely performed by means of the plasma disappearance of [^99m^Tc]-DTPA using a two sample method. Plasma samples were taken at about 120 and 180 min after injection of approximately 3 MBq [^99m^Tc]-DTPA [9]. DTPA-derived values were previously shown to yield an estimate of the GFR within 5% of the true GFR [10].

#### b) Relative renal function using [^99m^Tc]-MAG3

Renal split function (in %) was assessed by means of dynamic renal scintigraphy after intravenous injection of approximately 100 MBq [^99m^Tc]-MAG-3. Data were analysed according to the German procedure guidelines, by means of a region of interest (ROI) analysis with renal and subrenal background ROIs. The area under the curve in the time interval between 1 – 2 min after radiotracer injection was calculated in order to obtain the relative renal function [11].

### Data analysis

For statistical analysis the Pearson correlation coefficient and a Bland-Altmann statistic analysis were calculated (MedCalc, Version 12.7.8, Ostend, Belgium) as the purpose of the study was to determine the accuracy of single kidney GFR-measurement by using dynamic CTA.

## Results

All 7 patients (6 female; 58±7 years, range 47 – 70) had normal creatinine-levels (< 1.2 mg/dL). The mean body weight of the investigated patients was 75±9.4 kg (range 63 – 90), the mean BMI 28±2.9 (range 27 – 32). All acquisitions were performed without complications and no subject was excluded from the analysis. The patients were examined between August 2009 and February 2011.

**Figure 5:**
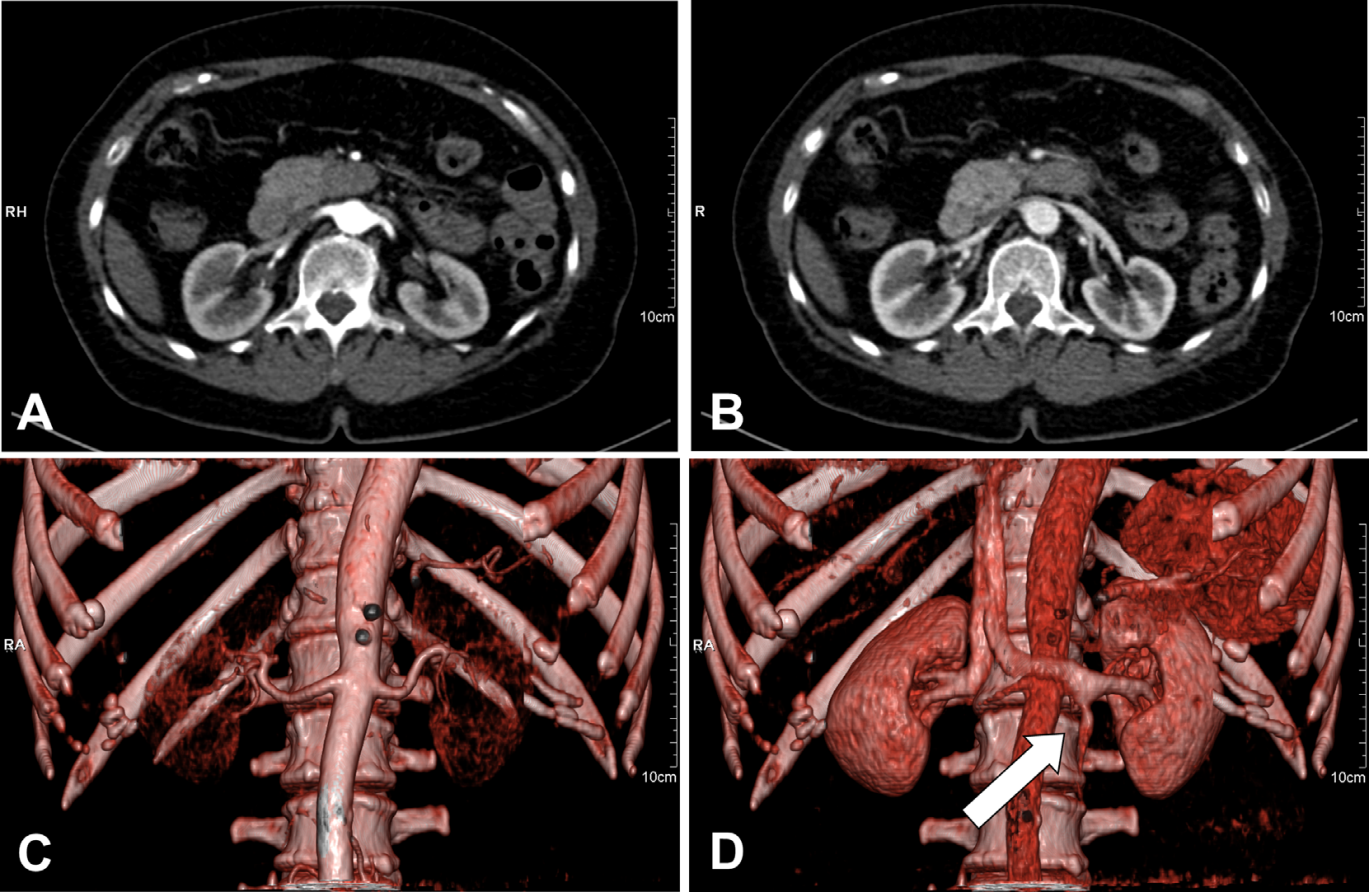
Arterial and venous scan after injection of 30(A, B) and volume rendering technique (C, D) with good identifiability of the abdominal aorta and renal anatomy. Note the ovarian vein (arrow in D).

**Figure 6:**
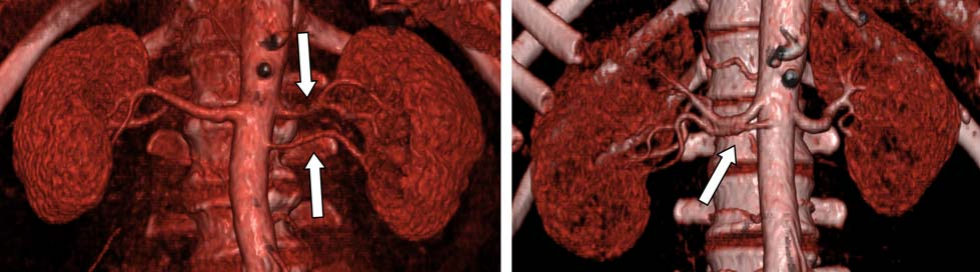
Anomalies of renal vessels; double renal arteries (arrows) in VRT.

The mean maximum attenuation of the abdominal aorta was 464 ±58 HU, of the renal arteries 435±48 HU, and the renal veins 277±29 HU. CNR was 18±0.4 and image noise was 25±6.2. The abdominal aorta and all renal vessels were depicted excellently (grade 1.0). The image quality score for cortex differentiation was 1.6±0.49, for the renal parenchyma 2.4±0.49. The effective dose was 8.96 mSv. Fig. 5 shows an example of an arterial and venous scan after injection of 30 mL contrast medium in cross sectional and volume rendering technique (VRT) with good identifiability of the abdominal aorta and renal anatomy. Double renal arteries were observed in two patients and are shown in Fig. 6.

**Figure 7:**
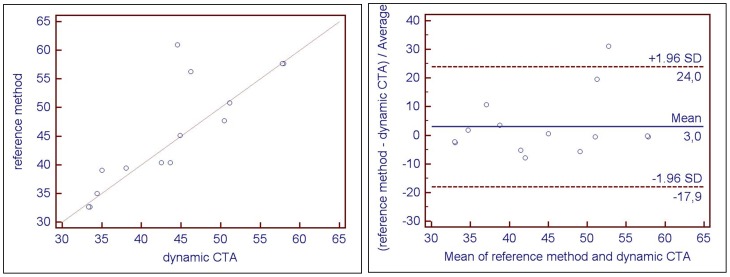
Statistical analysis performed using pearson correlation coefficient on the left (x- and y-axis show GFR in ml/min) and a Bland-Altman analysis on the right (x-axis shows GFR in ml/min, y-axis shows the difference between GFR values obtained using CTA and reference standard in %). Good correlation with a correlation coefficient of r = 0.84 (p = 0.0002). The Bland-Altman graph shows good agreement between GFR derived from dynamic CTA and GFR derived by reference method with a systematic error of only 3%.

Single kidney GFR was evenly distributed intraindividually with a maximum deviation of 5% (1.85±1.95%, range 0 – 5%). We did not observe detrimental motion effects caused by the continuous periodic table transport and the motion correction of the VPCT-body software sufficiently adjusted motion artifacts caused by respiration. There was a positive correlation between the mean GFR (n = 14) calculated by dynamic CTA (43.8±8.4 mL/min, range 33.4 – 58.0 mL/min) and the GFR obtained with the reference standard method (45.4±9.4 mL/min, range 32.6 – 60.9 mL/min). Fig. 7 shows the respective graph from the correlation analysis and the Bland-Altmann test with a correlation coefficient of r = 0.84 (P = 0.0002) and a systematic error of mean GFR of 3% (limits of agreement: – 17.9% to + 24%), respectively. The linearity of the Patlak fit for the cortical areas was very good, the square of the correlation coefficient always exceeded 0.9.

## Discussion

Accurate measurement of split renal function is indispensable in the preoperative evaluation of living donors as well as in patients eligible for the therapeutic regimen of many kidney diseases. In a previous study we simultaneously assessed morphology and function of renal transplants using dynamic CTA [4] and this time aimed to transfer this technique to patients with native kidneys. We again observed a good correlation (r = 0.84) of the GFR derived by dynamic CTA with our reference standard and only a small systematic error in the Bland-Altman test of 3%. Given the small sample size, the range between the limits of agreement appeare adequate. In contrast to our previous study (GFR by dedicated creatinine based formulas as standard of reference) the standard of reference this time was renal scintigraphy. Renal scintigraphy is considered to be even more accurate than using dedicated creatinine based GFR-formulas [12] and has not been used to validate the proposed dynamic CTA protocol so far. The average absolute deviation was only −0.5 mL/min and in contrast to our previous results, this time no overestimation of single kidney GFR was observed. This confirms the assumption that overestimation of GFR in the previous study has been caused by an increased interstital space due to massive impairment of the renal transplant function [4], [13]. We again applied the dynamic CTA protocol with a total scan time of about 40 seconds covering the renal vasculature as well as renal parenchyma. Due to the radiation dose exposure CT protocols generally cannot - in contrast to renal MRI perfusion protocols – simply be extended up to 5 min to cover the wash-out of the tubuli [14]. However, it has been demonstrated, that the Patlak model enables GFR-measurements even in case of a restricted time window [15]. The proposed CTA protocol provided good to excellent depiction of renal anatomy and therefore seems to be appropriate for evaluation of potential renal donors as well as assessment of various renal diseases. The multiphase technique furthermore allows to keep the amount of contrast medium at a very low level [16] which minimises the risk of contrast-induced nephropathy [17], which is especially advantageous in case of renal insufficiency.

The average dose of the proposed protocol was 8.96 mSv and in contrast to our previous studies (renal transplants in the pelvic region) [4], [16] less beam hardening artifacts occurred allowing for reduction of tube voltage to 80 kV even in overweight subjects. Yet although the average dose of the proposed protocol was 8.96 mSv and therefore lower than a standard biphasic CTA [5], [18], alternative and less invasive imaging techniques should always be considered. Contrast-enhanced MRI as an alternative modality that has the potential to measure split renal function [19] and as well facilitates simultaneous evaluation of renal anatomy and function [20]. Since patient with renal diseases often suffer from impaired renal function and application of gadolinium chelats in this particular patient population can cause nephrogenic systemic fibrosis [21] the risk of NSF always has to be balanced against the risk of CIN. New non-contrast MRI perfusion techniques like arterial spin labeling (ASL) appear to be a very promising alternative [22], [23], but are not yet able to reliably quantify GFR and more research is needed to validate these new technique [24].

Furthermore, contraindications against MRI (intracorporal devices, claustrophobia, incompliant and unstable patients) as well as the limited availability of MRI often restrict the application of these techniques in clinical routine. Hence, dynamic CTA protocols, which can be used for imaging of vascular, oncological and parenchymal diseases [25]–[27] seem to be a promising approach to improve patient management.

A limitation of our study is the small number of included subjects, which did not feature any renal pathology and further studies including more patients are necessary. However, the feasibility of assessing renal morphology and function in impaired kidneys using the proposed dynamic CTA protocol has separately been evaluated in a previous study [4] and the results are being confirmed by the current study using a more accurate reference standard (renal scintigraphy versus creatinine-based formulas). Furthermore, the concept of simultaneous assessment of renal anatomy and function has been applied successfully to patients with native kidneys.

## Conclusion

The proposed dynamic CTA protocol enables to comprehensively assess renal anatomy and function using a single-stop CT-based examination and is therefore a powerful non-invasive tool for renal diagnosis.
